# A site suitability analysis for castor (*Ricinus communis* L.) production during Brazil's second harvest incorporating disease prediction

**DOI:** 10.1016/j.heliyon.2023.e18981

**Published:** 2023-08-06

**Authors:** Travis W. Witt, K. Colton Flynn, Tiago Zoz, Trey O. Lee, José E.B. A. Monteiro

**Affiliations:** a USDA-ARS, PA, Grazinglands Research Laboratory, 7207 West Cheyenne Street, El Reno, OK, 73036, USA; b USDA-ARS, PA, Grassland Soil and Water Research Laboratory, 808 East Blackland Road, Temple, TX, 76502, USA; c UEMS, Center for Studies and Innovations in Carbon Sequestration (CEISCO), BR163 - km 20,2, Mundo Novo, MS, 79540-000, Brazil; d EMBRAPA Informática Agropecuária, 209 André Tosello Street, Campinas, SP, 13083-886, Brazil

**Keywords:** Site suitability analysis, Gray mold, Off-season, Oilseed crop

## Abstract

Castor (*Ricinus communis* L.) is an important industrial crop with a wide range of industrial and pharmaceutical applications. Brazil is among the largest castor-producing countries. Between 2004 and 2010, castor cultivation was stimulated with an emphasis towards biodiesel production. However, this was not enough to leverage the production of castor in Brazil, mainly due to the lack of structured trade and the competition with other cheaper raw materials for the production of biodiesel. Despite this failure, the species presents itself as an excellent alternative for crop rotation in the second crop among soybean, corn, beans, and cotton cultivation areas as the oil is highly valuable for other products. Moreover, it has drawn the attention of producers and researchers in Brazil for this potential rotation as it is considered a plant tolerant of water-deficiency and is highly susceptible to gray mold, a disease favored by high humidity in the final stages of the crop. For instance, its cultivation in the second crop in Cerrado regions, where rains occur in the early stages of the crop and cease when the plants reach the final stage of production, has been successful and shows great promise. The current study aimed to evaluate the suitability of environments throughout Brazil to grow castor, incorporating variables associated with the incidence of gray mold and confirm these findings based on existing castor trial data obtained from the literature. The site suitability analysis determined that 74.99 million hectares - 8.8% of Brazilian territory - are highly suitable for castor production during second harvest, mostly located in the Northeastern and Midwestern regions. These results are surprising since Brazil currently has around 7.8% (∼66.81 million hectares) of its territory occupied with agriculture (grains, fruits, vegetables, and perennial crops). The findings of this study provide a method to perform site suitability for crops using data associated with agronomic and disease characteristics, as is the case with gray mold that often results in significant losses in castor production. Also, this analysis provides evidence for the great potential of Brazil to increase castor production and meet the world demand for its oil through utilization of second-crop cultivation.

## 1 Introduction

The current global scenario has highlighted the search for alternative sources to petroleum products. In this context, castor (*Ricinus communis* L.), an oilseed crop, could serve as an alternative oil with unique characteristics. Castor oil has a wide range of industrial applications, such as lubricants [1], coatings [2], plastics [3], and adhesives [4]. Chemical compounds extracted from castor leaves, roots, grains, and stems also have numerous pharmacological applications such as cytotoxicity, antibacterial, anti-asthmatic, anti-inflammatory, and other medical applications [5].

There has been great incentive for castor production in Brazil and other countries in recent years, with intention mainly towards the production of biodiesel [[6], [7], [8], [9]]. Due to these interests, some cultivars adapted for mechanized cultivation under crop rotation systems were developed. These cultivars are grown in Brazil's second crop, mainly after soybean (*Glycine max*) or corn (*Zea mays*) grown in the summer [9]. Among other factors, the occurrence of high rainfall during the ripening period favors the occurrence of gray mold (*Amphobotrys ricini* (Buchw.) Hennebert) that leads to significant yield losses when castor is grown in the first crop (from September to January). Temperatures in the range of 20–25 °C, high relative humidity, and the occurrence of rainfall are favorable to the occurrence of gray mold [10]. Therefore, Brazil's castor cultivation must be carried out in the second crop - from January to August - in which there are rains in the initial stages of crop development, and subsequently it ceases during the maturation stage of the castor, thus, avoiding gray mold occurrence.

Gray mold is considered the most damaging plant pathogen of castor plants [11]. The flowering and fructification stages are the plant development stages most susceptible to the fungus. Symptoms of the fungus are observed on the inflorescences and fruit bunches as grayish or bluish spots; in the fruits, the symptoms are circular or elliptical, sunken, dark-colored spots, which lead to rupture or rotting of the capsule and grains [12]. Additionally, exposed lesions increase and become grayish-brown blights, with the occurrence of a large conidial mass in humid conditions, which are favorable for disease development [13].

Despite the boost in castor production in Brazil, its suitability for cultivation lacks geospatial information. A study on suitable areas for the cultivation of castor considering the edaphoclimatic characteristics (climate and soil) and the potential for the occurrence of disease - gray mold - is essential to support Brazilian producers and researchers. Also, as in other crops, the genotype x environment (G x E) trials in the developing process of cultivars are expensive and can take years to conduct. An alternative to reduce costs and time associated with G x E is the use of a geographic information systems (GIS) approach, specifically site suitability analyses. Such an approach can be a helpful tool to identify mega-environments, thus limiting the number of G x E trials that a plant breeder would need to evaluate [14], consequently resulting in a reduction of costs.

The identification of mega-environments can reduce the need for many of the test locations for G x E studies as shown for cotton production in the US cotton belt [15]. Similar research [14] has successfully identified locations in the US for castor to expand while considering the potential for loss in production due to disease. Such research is important for the expansion of castor production in Brazil to increase awareness of castor production potential among farmers, stakeholders, and policy makers.

Given the above, this study has two objectives: 1) evaluate the suitability of environments throughout Brazil to grow castor incorporating multiple geophysical/geochemical variables as well as the variables associated with the incidence of gray mold; and 2) confirm these findings based on existing Brazilian castor trials obtained from the literature.

## 2 Materials and methods

### 2.1 Study area

Castor has been grown throughout Brazil for decades; however, with the development of dwarf hybrids and mechanical production, there is a desire to revisit suitability, especially as a second crop. The current study area consisted of the 26 states of Brazil, an area of ∼8.5 million km^2^. However, due to a lack of data near the national border some areas were not included in the analyses. Brazil has a wide range of climates, with approximately 81%, 5%, and 14% within the Köppen climate classifications of A (tropical), B (dry), and C (humid subtropical), respectively [16], and within these climates are a variety of soil types and geophysical characteristics within each of these climates [17]. The climate and soil and geophysical characteristics evaluated in the current site suitability study are listed in Table 1.

**Table 1:** Rankings of each criterion for analytical hierarchy process (AHP) calculations from January 1 to August 30.

Main Criteria	Sub-criteria	Ranking
Average Precipitation (mm) *January to August*	>1000 (>39.4 in) 800-1000 (31.5 in-39.4 in) 600-800 (23.6 in – 31.5 in) 400-600 (15.7 in – 23.6 in) 200-400 (7.9 in – 15.7 in) <200 (<7.9 in)	8 10 8 7 5 1
Average Temperature (°C) *January to August*	20–30 >30 16–20 <16	10 7 5 1
Evapotranspiration *January to August*	≤ total precipitation > total precipitation	10 1
Soil pH	5–8 8–10 4–5 <4	10 7 5 1
Soil Texture (% Clay)	15–25 25–40 40–50 <15 >50	10 8 6 2 1
# of Rainy Days (Disease Prone)	<30 30–60 60–75 75–90 90–105 105–120 120–135 135–150 >150	10 8 7 6 5 4 3 2 1
Temperature (°C) (Disease Prone)	<15 15–20 20–25 25–35	10 8 2 1
Land Cover	11, 14, 18, 19, 21, 24 Other Categories	10 1

### 2.2 Soils and geophysical data

Soil and geophysical data (pH, clay content, soil drainage, and land cover) are important for determining where castor can be grown. The soil data of pH, clay content, and soil drainage were obtained from GeoInfo using data from only the 0 to 5 cm range [18]. Soil elevation data were obtained from the ASTER Global Digital Elevation Model v003 [19]. In the current study, soil texture was considered the most important factor for castor production (Table 2). Soil texture was considered the most important factor, because it influences water holding capacity, the ability to adjust the pH through liming, and nutrient uptake through cation exchange capacity (CEC) and anion exchange capacity (AEC). Past research has shown that castor performs best in well-drained soils. Castor growth is best suited to soils with good drainage [20,21] and high pH soils; however, castor can tolerate soil pH values between 5 and 8 [22]. The second most important factor was soil pH. Although soil pH can easily be adjusted through the addition of calcium carbonate or calcium sulphate this would be an additional input cost for castor producers. Additionally, many soils in Brazil have a low pH which leads to aluminum toxicity. Land classification data were obtained from the Oak Ridge National Laboratory Distributed Active Archive Center (ORNL DAAC) [23] using all land cover types with the term “agriculture”. However, forested lands were purposefully excluded, to not encourage deforestation. Land suitability classifications were given suitability rankings based on other suitability studies [14,24]. Land cover was considered the least important factor (ninth) in this study, because castor will be grown as the second crop (i.e. already on land suitable for crop production). These soil and geophysical requirements found in past literature for castor served as the basis for the criterion ranking for the soil/geophysical variables (Table 1, Table 2).

**Table 2:** Pairwise comparison matrix.

Criteria	Soil Texture	Soil pH	Avg Temp.	Avg Precip.	ET	Disease Temp.	Disease Precip.	Land Cover
Soil Texture	1	2	3	4	4	6	6	9
Soil pH	1/2	1	3	4	4	6	6	9
Avg. Temp.	1/3	1/3	1	2	2	4	4	6
Avg. Precip.	1/4	1/4	½	1	1	3	3	6
ET	1/4	1/4	½	1	1	3	3	6
Disease Temp.	1/6	1/6	¼	1/3	1/3	1	1	4
Disease Precip.	1/6	1/6	¼	1/3	1/3	1	1	4
Land Cover	1/9	1/9	1/6	1/6	1/6	1/4	1/4	1

Avg. Temp. – Average temperature; Avg. Precip. – Average precipitation; ET – Evapotranspiration; Disease Temp. - Temperature prone to disease; Disease Precip. - Precipitation prone to disease.

### 2.3 Climatic data

Castor is native to tropical and subtropical regions of the world. However, like any plant, it is limited by environmental factors. Castor is often grown as the second crop in Brazil (January to August); this period usually has less favorable conditions for crop growth than the first cropping period. Climatic data is important for determining the areas that may be suitable for castor production during the second cropping season. Climatic information was obtained from the meteorological databank of the National Institute of Meteorology (INMET) [25]. Data representing the period from January 1st to August 31st, for the years 2000 to 2014, were downloaded for all available automatic weather stations (n = 617); however, due to some stations (n = 175) not having data for this period and/or having less than ten years of data they were not incorporated. INMET has weather stations spread throughout the country with a good density in all regions except the northwest (Amazonian biome), with each state having several weather stations. The climatic data was considered the third and fourth most influential factors for castor production during the second cropping season. The average temperature was the third most important factor, because castor needs certain temperatures for germination, flower initiation and fertilization, and seed maturation. Castor requires a base temperature of 15.6 °C for germination [26], with 25 to 31 °^◦^C as the optimum temperature [27]. Long periods of temperatures above 30°C can limit female flower production, and temperatures above 40 °C will limit plant growth [28,29].

The fourth most important factor for castor production is average precipitation. The second cropping season in Brazil is very dry with little rainfall occurring after the month of April. Castor produces high-quality seeds when annual rainfall is approximately 1,360 mm [20,30]. Although supplemental irrigation of castor is possible many of the farms in Brazil do not have irrigation systems. These climatic requirements found in past literature for castor served as the basis for the criterion ranking for the climatic variables (Table 1, Table 2).

### 2.4 Abiotic and biotic limitations

Two main factors limiting castor production are the evapotranspiration rate and the presence of gray mold. Evapotranspiration (ET) was also considered the fourth most important trait. ET is driven by both the average temperature and the average precipitation. The transpiration part of ET drives plant production; however, it is very difficult to separate the ET into evaporation and transpiration [31]. Evapotranspiration is essential for predicting plant growth and possible plant stress. If evapotranspiration exceeds precipitation, castor plants will close their stomata which limits CO_2_ uptake and, ultimately limits yield [32]. Evapotranspiration data were obtained from monthly reference evapotranspiration for Brazil from Zenodo. The reference ET data on Zenodo was previously published [33]. Dias et al., 2021 [33] calculated reference Et using a modified Penman-Monteith method [[34], [35], [36]] and weather data from INMET. Gray mold is the number one pest of castor production worldwide. Many factors increase or decrease the prevalence of gray mold in a castor field. However, the factors that contribute the most to gray mold growth is when temperatures are between 14.6 and 28 °C and 72 h of rainfall [37]. Disease temperature and precipitation were considered the sixth most important factors for castor production due to the possibility of Gray mold not occurring when conditions are ideal and the possibility to use fungicides or to breed resistant castor cultivars in the future. Each of these abiotic/biotic characteristics were found in past literature for castor and served as the basis for the criterion ranking for the abiotic/biotic limitation variables (Table 1, Table 2).

### 2.5 Calculation of weights and map production

The criteria used in castor production were ranked from 1 to 9 (in order of increasing importance). The ranking was based on using the authors’ experience with the crop and a review of previous studies [24,38]. Using these ranks, the analytical hierarchy process (AHP) was applied to calculate normalized weights of the rankings for each factor [38,39]. Consistency among the methods to ensure minimal bias involved the use of a combination of the random consistency index (RI) and a consistency index (CI), the number of criteria in the study (n = 8), and the consistency ratio [24,38]. The CI was calculated using the following formula:

CI = (*λ*_max_ – n)/(n – 1) = 0.054

Where CI denotes the consistency index, *λ*_max_ is the principle eigenvector of the matrix, and n is the order of the matrix.

The process of the AHP provides weights for each of the criteria that is based on past literature and professional insights. These weights are used in conjunction with sub-criteria rankings to determine a suitability ranking. Suitability rankings for each pixel of the area of interest determine the site suitability of the given location from one (least suitable) to ten (most suitable). Using the sub-criteria ranked layers and AHP calculated weights, the following equation is used to determine each pixel's suitability ranking via a weighted overlay methodology:

$$SS=\sum_{i=1}^{n}W_{i}X_{i}$$

Where SS denotes the site suitability score, *W*_*i*_ is the weight for the individual criterion being measured, *X*_*i*_ indicates the sub-criteria value, and n is the total number of criteria (n = 8) [40]. The synthesized matrix for relative weights can be in Table 3. Following the calculation for each 30 m pixel across the whole country of Brazil, the site suitability measures were categorized into suitability levels as suggested by the Food and Agriculture Organization of the United Nations (FAO) that included [1]: Highly suitable [10-8] [2], moderately suitable [8–6] [3], marginally suitable [6–4], and [4] currently not suitable [4–2] [24,41]. Rendering geospatial representation determines where castor is most suitable to be produced in Brazil. The geospatial analytics among the raster layers representing sub-criteria rankings and eventual weighted overlay analytics were conducted in ArcGIS 10.8 utilizing the raster calculator data management tool [42].

Max. eigenvalue (*λ*_max_) = 8.375.

N = 8.

Consistency index (CI) = (*λ*_max_ – n)/(n – 1) = 0.054

Random index (RI) = 1.41.

Consistency ratio (CR)=(CI/RI) = 0.038

**Table 3:** Synthesized matrix for relative weights.

Criteria	Soil Texture	Soil pH	Avg. Temp.	Avg. Precip.	ET	Disease Temp.	Disease Precip.	Land Cover	Weights
Soil Texture	0.360	0.468	0.346	0.312	0.312	0.247	0.247	0.200	0.316
Soil pH	0.180	0.234	0.346	0.312	0.312	0.247	0.247	0.200	0.265
Avg. Temp	0.120	0.078	0.115	0.156	0.156	0.165	0.165	0.133	0.136
Avg. Precip.	0.090	0.058	0.058	0.078	0.078	0.124	0.124	0.133	0.090
ET	0.090	0.058	0.058	0.078	0.078	0.124	0.124	0.133	0.090
Disease Temp.	0.060	0.039	0.029	0.026	0.026	0.041	0.041	0.089	0.042
Disease Precip.	0.060	0.039	0.029	0.026	0.026	0.041	0.041	0.089	0.042
Land Cover	0.040	0.026	0.019	0.013	0.013	0.010	0.010	0.022	0.019

Avg. Temp. – Average temperature; Avg. Precip. – Average precipitation; ET – Evapotranspiration; Disease Temp. - Temperature prone to disease; Disease Precip. - Precipitation prone to disease.

### 2.6 Accuracy analyses

Independent and dependent data were analyzed in SAS Studio 3.8 [43] using the orthoregression procedure (general linear model by least squares using the orthogonalization method of Gentleman-Givens transformation). Analyses were performed to validate the accuracy of the site suitability analyses. Yield data were obtained from the years 1980 to 2018 from experiments conducted by researchers across Brazil [[44], [45], [46], [47], [48], [49], [50], [51], [52], [53], [54]]. These yield data were compared to the site suitability value of the specific geographic coordinates and average site suitability values of a 1-km radius buffer from those same coordinates. Additional regression analyses, employing the use of R^2^ values as coefficients of determination, were performed on data for the years 2000–2014 for the first (September to December) and second (January to August) seasons to evaluate the accuracy of the model across growing seasons. Additionally, the source of genetic variability (cultivar vs. hybrid) was evaluated for 1980–2018 by performing regression analyses. The regression analyses compared the yield from the different sources of genetic variability (cultivar or hybrid) to the site suitability values and the 1-km radius buffers. The R^2^ value for the hybrids was higher than the cultivars for the regression analyses, which indicated that the hybrids fit the site suitability model better and would be better adapted to the differing growing conditions across Brazil.

## 3 Results

### 3.1 Soils and geophysical data

**Fig. 1:**
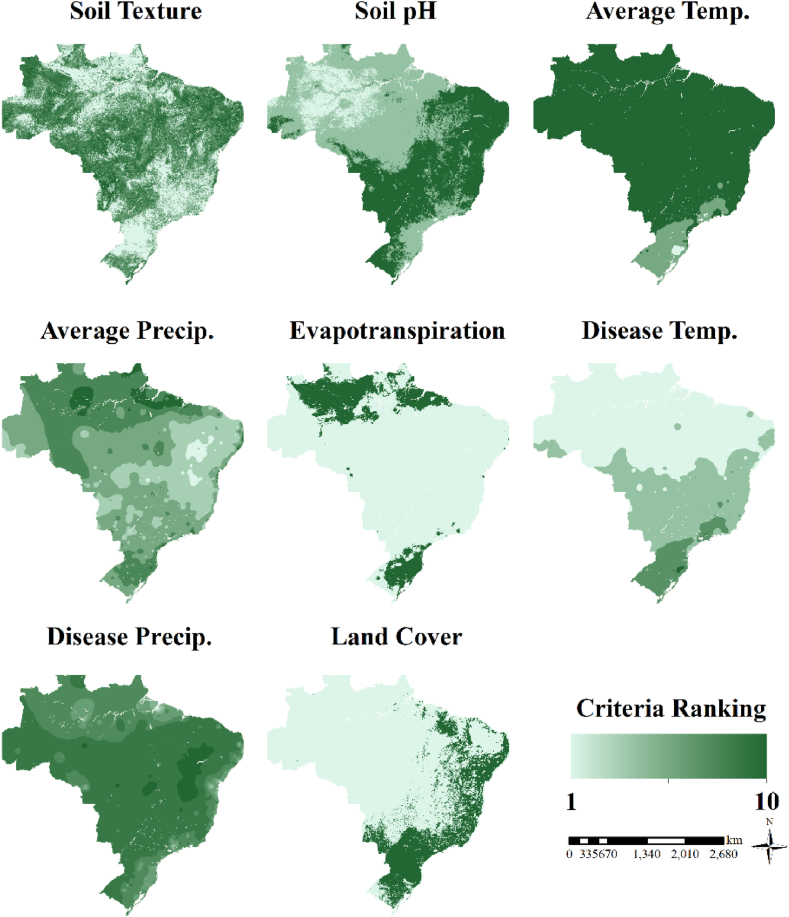
Average 15-year suitability maps for each criterion. Light areas represent low suitability [1], and dark areas represent high suitability [10] for each criterion. The maps show Soil Texture, Soil pH, Average Temp. – Average temperature; Average Precip. – Average precipitation; Evapotranspiration (Et) Disease Temp. - Temperature prone to disease; Disease Precip. - Precipitation prone to disease.

Regarding soil pH, 41.36% of Brazilian hectares are considered highly suitable for castor production, covering the Midwest, Southeast, Northeast, and part of the South (Fig. 1). No occurrence of soils with a pH greater than 8.0 was observed. Brazilian soils are usually acidic, with a predominance of negative charges. Still, considering the pH of the soil, 48.59% of areas are considered marginally suitable for castor production, and 10.05% are currently not suitable for castor production (Fig. 1).

Regarding soil texture, 51.78% of the territory has a clay content of between 15 and 40%, which is considered highly suitable for castor production. On the other hand, 30.49% of areas are considered currently not suitable for castor production. For the land cover, 21.98% of Brazil is highly suitable, and 78.02% is permanently unsuitable (Fig. 1).

### 3.2 Climatic data

For the climatic data, 3.56% of Brazil has average precipitation between 800 and 1000 mm, and 24.11% between 600 and 800 mm or >1000 mm. Therefore, 27.67% of areas are highly suitable for castor production, based on precipitation. On the other hand, 0.95% of the territory has average precipitation lower than 200 mm, which is currently not suitable for castor production. This area is located mainly in the interior of the northeast region of Brazil (Fig. 1).

Regarding average temperature, 84.34% of the area is highly suitable for castor production, and 0.18% is currently not suitable for castor production. It is worth mentioning that there are no areas with an average temperature higher than 30 °C in Brazil, and 15.46% of the area has an average temperature between 16 and 20 °C. For evapotranspiration, in 16.41% of the area, the evapotranspiration does not exceed the precipitation and therefore is highly suitable for castor production. This area is located in the North and South regions of Brazil (Fig. 1). In 83.59% of the territory, evapotranspiration exceeds the total precipitation, which may lead to plant stress.

### 3.3 Disease pressure

Gray mold occurs when both precipitation and temperature are favorable for its growth, so disease ratings are based on the ability to avoid the disease in each location. For the effect of precipitation on the disease occurrence, 65.05% of the area had the least precipitation needed for disease growth (highly suitable for no disease growth). This area is located mainly in the interior of the northeast region of Brazil, a region characterized by low average precipitation over the year. Only 0.10% of Brazilian territory is classified as highly favorable for disease growth. These areas coincide with the coast and parts of the Amazonian region (Fig. 1).

Regarding the influence of temperature on gray mold growth, 15.66% of the territory is considered highly suitable for no disease growth. These areas are characterized by a higher latitude, as in the case of the South region, or by mountain regions with higher altitudes, as observed in parts of the Southeast. On the other hand, 41.48% of the territory is classified as highly favorable for disease growth, mainly located in the North and Northeast regions (Fig. 1).

### 3.4 National-level site suitability

**Fig. 2:**
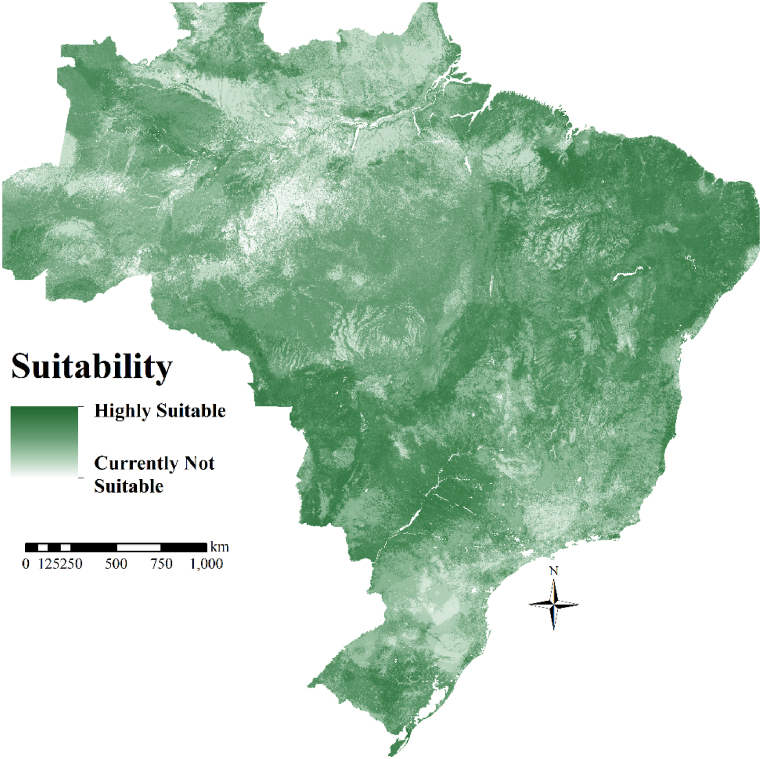
Site suitability (15-year average) of Brazil.

Regarding the national-level site suitability, the regions with the highest suitability for castor production in Brazil, considering the second harvest, are east and north of the Northeast, south and west of Midwest, west of São Paulo and Minas Gerais, and south of Rio Grande do Sul, comprising the total of 74.99 million hectares (Fig. 2). Brazil has around 428 million hectares considered moderately suitable for castor production (Fig. 2). Our study shows that Brazil does not have areas considered permanently not suitable for castor production, though there are some that are currently not suitable (Fig. 2).

### 3.5 Accuracy assessment

Overall, the site suitability analyses performed well compared to yield data of past castor trials (1980–2018) in Brazil. All regression analyses were highly significant (*p* ≤ 001). There was an R^2^ value of 0.50 between the site suitability analyses and a specific (point data) castor trial's yield data. However, at a one km radius from the point the R^2^ value increased to 0.54. This indicates that the site suitability analyses is more accurate for a region then a specific farm, which may relate to the original spatial resolution of the various layers. For example, a field trial was conducted at Adamantina, SP, Brazil. The site suitability for Adamantina was determined to be 7.87, but at a one km radius from Adamantina then the site suitability was 7.60. When the site suitability analyses was evaluated for the first or second harvest yield data for 2000–2014 at a specific point, then there was an R^2^ of 0.29 for the first season and an R^2^ of 0.62 for the second season. At a one km radius, the R^2^ increased to 0.42 for the first season and 0.64 for the second season. The low R^2^ values for the first season may be due to an over prediction of gray mold. The first season usually has environmental conditions suitable for gray mold growth. However, the trial data did not specify if gray mold was observed. The R^2^ value for cultivars was 0.46 for the specific point, but it was 0.96 for hybrid production. At a one-km radius the R^2^ for cultivars increased to 0.51, but the hybrids remained at 0.96. These results indicate that the short statured hybrids are more adapted to different environments and should be used in future castor production.

## 4 Discussion

To contextualize the territorial dimensions of Brazil, the country has a total area of 851.6 million hectares, of which 66.3% is covered by native vegetation, 30.2% is occupied by agriculture (21.2% livestock, 1.2% planted forests, and 7.8% production of grains, fruits, vegetables, and perennial crops), and the remaining 3.5% are for urban use [55]. For the site suitable analysis, according to the present study, Brazil has 74.99 million hectares classified as highly suitable for castor production. However, currently (2021/2022 harvest), Brazilian castor production occupies 48.9 thousand hectares, equivalent to 0.07% of the potential area [56].

The analysis suggests some areas with the highest suitability for castor production in Brazil coincide with areas of traditional castor production, such as the Northeast region. In the last harvest (2021/2022), the Northeast was responsible for 98.2% of national production and occupied 98.4% of the castor cultivation area. Also, in this region, only two states currently present significant cultivation areas, Bahia and Ceará, with 97.4% and 1.0% of the Brazilian cultivation area, respectively [56].

In addition to the Northeast, other areas were also classified as highly suitable for castor production in the second harvest incorporating disease prediction. Our study showed great potential to increase castor production on a large scale in the Midwest since a significant area of Brazil, classified as highly suitable for castor production, is located in this region. Also, west of São Paulo and Minas Gerais, northwest of Paraná, and center and south of Rio Grande do Sul also have areas considered highly suitable for castor cultivation (Fig. 2). In most of these regions, soybean and corn are grown in the first harvest. Therefore, our study suggests castor cultivation after these crops.

Brazil has two contrasting castor cultivation systems. One is the traditional system, carried out in small areas, characterized by the low use of technology, often intercropped [[57], [58], [59]] with other species, and with the use of genotypes not adapted for mechanized harvest. The other system has been growing in recent years and is carried out in large areas, in most cases, traditionally used for soybean and corn cultivation. This system uses high technology with mechanized operations [60]. Another characteristic is the use of adapted hybrids for mechanized harvesting, exploiting the heterosis of the species [9].

Our study shows that Brazil has an enormous potential to increase castor production without the need to clear new areas. In a significant part of the areas considered highly suitable for the castor cultivation, agriculture is already carried out; that is, it would be easier to start the cultivation of castor since small changes in the production system, especially in agricultural areas, would be enough to start mechanized castor cultivation compared to opening new areas. In addition, as already highlighted, due to the lower occurrence of diseases, castor obtains higher yields when cultivated in the second crop in the Midwest and Southeast of Brazil, after the summer crop, which is usually the most profitable, therefore, the adoption of its cultivation by producers is easier since it is not competing with the currently most profitable crops on farms.

It should also be noted that the existence of marginal areas, which due to some factors, such as the degree of degradation, distance from commercial centers, lack of logistics, or other factors that do not make it interesting for agriculture, are not intensively used. These areas are usually cheaper to lease or acquire, serving as potential candidates, once corrected, for the expansion of castor cultivation. Brazil has around 154 million hectares occupied with pastures, of which 53% are degraded or need some correction [61]. Most of these degraded areas are located in the states of Mato Grosso, Mato Grosso do Sul, São Paulo, Minas Gerais, Paraná, Goiás, Tocantins, and Bahia, and according to the site suitability analysis, part of these areas are considered highly suitable for castor production. In these areas, there is no competition with other crops; therefore, they are potential areas that can be recovered with castor cultivation.

However, some challenges should be overcome. First, due to little publicity and knowledge, some producers of other crops consider the species a weed. Another key factor in encouraging cultivation is structuring the castor market in Brazil [62]. Producers from other regions that are not traditionally involved in castor cultivation are often unaware of companies that would buy the castor grain produced. In addition, there are few advances regarding the mechanization of its cultivation, mainly related to crop harvest [62]. Equipment used for mechanized harvest of castor has had losses between 247 and 404 kg ha^−1^, equivalent to 16% and 21.2% of total production [63], considered high compared to other crops, like soybean and corn, which has an acceptable loss in the harvest process 60 and 90 kg ha^−1^, respectively [64,65].

Other technical difficulties can also be highlighted. For example, the development of castor genotypes adapted for large-scale cultivation with mechanized harvesting is still slow. In some cases, the genotypes cultivated in Brazil are imported from other countries and not necessarily completely adapted for cultivation in Brazil. Currently, in the National Cultivars Registry (Registro Nacional de Cultivares - RNC), there are 54 registered castor genotypes (adapted or not for mechanized harvesting); 10 are parental lines, and 44 are cultivars [61]. These genotypes are in possession of 11 organizations, four of which are public research agencies and seven companies. These statistics show that the development of castor cultivars in Brazil is much lower than the main crops grown in Brazil, such as soybean and corn, which have 4,571 and 6,552 genotypes registered, respectively, in the RNC [61].

Another critical challenge to increasing castor production in Brazil is the control of gray mold, the main disease that causes damage to the castor crop (Lourenço et al., 2018). Our findings are innovative in that they consider not only edaphoclimatic characteristics in the site suitable analysis but also the potential for the occurrence of gray mold and second crop cultivation. We found only one study similar to this one with castor results for the United States and considering cultivation throughout the year [14]. These challenges are the key components responsible for the resistance by producers to grow castor in their areas. Cultivation of castor during the second crop would be important for Brazil as this time does not compete with the number one cash crop of the country (soybeans). Also, the current second crop for most of the country (maize) is being affected by a costly pest (maize leafhopper; *Dalbulus maidis*). If some producers switch to an alternative crop, such as castor then pest populations may decrease.

Also, due to taking into account the edaphoclimatic characteristics of Brazil and associating the risk of occurrence of the main disease that occurs in castor plants (gray mold), the results of the present study are valuable and will help castor breeding programs in Brazil. Before the commercialization of a genotype of any plant species as a cultivar, it is necessary to obtain information about genotype-environment interaction in experiments known as G x E trials. These experiments are essential to evaluate the stability and adaptability of the genotypes [66]. The locations for implementing the G x E trails must be carefully chosen to faithfully represent the edaphoclimatic cultivation conditions in the different production regions [67]. The number of sites for the implementation of G x E trials must also be carefully defined since a small number of sites may not efficiently represent the different cultivation conditions. On the other hand, a large number of sites can increase the costs of the breeding program and present redundant results. The results of this study show the different levels of suitability for castor production. This will allow castor breeders and geneticist to identify different regions to test their newly created germplasm. For example, central Brazil and southeastern Brazil may be good areas to test for tolerance to aluminum toxicity due to the unfavorable pH in these areas. Additionally, extreme southern Brazil does not have temperatures that are optimal for castor production during the second season, so a breeder may want to observe castor in this region for cold tolerance.

Despite the significant challenges, Brazilian castor production has enormous potential to increase due to the increasing global demand for castor oil and its derivatives. In this sense, the present study stands out for a series of benefits that could boost Brazilian production. It is the first study that covers the entire territory of Brazil and delivers a complete mapping of the most suitable areas for castor bean cultivation in the second crop, including considering the conditions prone to the occurrence of the main disease of the crop. Based on this study, public policies can be developed to encourage castor cultivation in suitable regions. In addition, insurance plans for castor bean crops, which currently do not exist in the country, may be structured. Another important aspect of the study is social and environmental preservation, as it highlights the possibility of significantly increasing Brazilian castor bean production, not competing with food crops and without the need to deforest areas. On the contrary, besides evidencing castor as a potential species in crop rotations systems, the study even shows possible degraded areas that can be recovered with castor cultivation, a rustic species that reaches satisfactory yield, which other species, like soybean or corn, would not reach in these areas. Also, another benefit is the possibility of castor breeding programs choosing suitable locations to conduct the GxE trials based on the results of this study, and this way, maximize the GxE interaction results, becoming more efficient in the development of castor genotype resistant to gray mold and under different edaphoclimatic conditions.

Despite all the already highlighted benefits of the study, the fact that it presents results for the entire Brazilian territory can be considered a drawback when we want to study the influence of edaphoclimatic characteristics on castor bean cultivation in specific microregions. Therefore, the next steps based on the results of this study involve the development of regionalized studies with higher resolution to provide more accurate information to increase Brazilian castor bean production. Additional drawbacks to site suitability studies are the need to use average temperatures and average rainfall. As noted previously, the extreme temperatures (<15.6 °C and >40 °C) are unfavorable to castor production, which were not necessarily identified when averages are used. Using averages may have over predicted the suitability for castor production especially closer to the equator (Northern Brazil) as these regions stay closer to 40 °C year-round. Rainfall distribution is important for all crops especially at plant establishment, flower initiation, and seed formation. Using average rainfall may not have accounted for this variability in rainfall distribution. Additional studies may be able to look at site suitability analyses for a much narrower range of conditions. For example, they may use weather forecasting models to anticipate above normal temperatures or below normal precipitation. Also, more agronomic trials are needed to better understand how castor responds to intermittent drought or intermittent high or low temperatures which will help in fine tuning site suitability analyses and ultimately new castor hybrids.

## 5 Conclusion

This study aimed to evaluate the suitability of environments throughout Brazil to grow castor by combining agronomic and climatic variables with gray mold forecasting to find the potential areas that Brazilian castor production could be established or improved. This study can help plant breeders to identify contrasting environments that allow for more valuable evaluations of new germplasm. The site suitability analysis performed in the current study could aid producers in identifying regions with more potential for castor production in Brazil, considering the cultivation in the second harvest. Additionally, the study shows that researchers and producers should pursue hybrid castor production in Brazil. This site suitability study forecasting had a good accuracy for the prediction of castor yields of hybrids when compared to historical data (2000–2014). The R^2^ was greater than 0.62 for castor production during the second season for all genotypes and was 0.96 for hybrid production. Although this site suitability study attempted to define the higher potential areas for castor production in Brazil castor production considering the potential occurrence of gray mold, we acknowledge that fungicides, breeding/genetics, and agronomic practices may be able to decrease the occurrence of disease, thus drastically changing the areas that would be suitable for castor production in the second harvest. Although the forecasting in the current study was highly predictive of castor production during the second harvest it could be improved by evaluating transportation and infrastructure limitations, the profitability of castor compared to other second season crops or use specific climatic conditions to predict castor production. For example, some areas of Brazil may receive the same average rainfall but have different distributions. However, the weather data collection especially through automated weather stations is still in its infancy throughout Brazil.

## Author contribution statement

Travis W. Witt, K. Colton Flynn and Tiago Zoz: Conceived and designed the experiments; Performed the experiments; Analyzed and interpreted the data; Contributed reagents, materials, analysis tools or data; Wrote the paper.

Trey O. Lee: Performed the experiments; analyzed and interpreted the data; Contributed reagents, materials, analysis tools or data; Wrote the paper.

José E. B. A. Monteiro: Analyzed and interpreted the data; Contributed reagents, materials, analysis tools or data; Wrote the paper.

## Funding statement

Travis Witt was supported by funding from USDA-ARS 301-3070-5050-3.

## Data availability statement

Data associated with this study has been deposited at Data from: A site suitability analysis for castor (Ricinus communis L.) production during Brazil's second harvest accounting for potential disease. Ag Data Commons. https://doi.org/10.15482/USDA.ADC/1528958.

## Declaration of competing interest

The authors declare that they have no known competing financial interests or personal relationships that could have appeared to influence the work reported in this paper.
