# Supplementary material for: A site suitability analysis for castor (Ricinus communis L.) production during Brazil's second harvest incorporating disease prediction
Source: Heliyon. 2023 Aug 6;9(8):e18981. doi: 10.1016/j.heliyon.2023.e18981 (PMC10432709; doi:10.1016/j.heliyon.2023.e18981)
Supplement: Multimedia component 1 [file mmc1.docx]

**Supplementary material**

Estimating of area (hectares and percentage) for castor cultivation in the second harvest in the Brazilian territory according to the sub-criteria of the site suitable analysis

| **Main Criteria** | **Sub-criteria** | **Area (ha)** | **Area (%)** |
| --- | --- | --- | --- |
| Average Precipitation (mm)  *January to August* | 800-1000  600-800; >1000  400-600  200-400  <200 | 59,165,603 400,979,105  959,331,776 228,117,134  15,760,083 | 3.56  24.11  57.67  13.71  0.95 |
| Average Temperature (°C)  *January to August* | 20-30  >30  16-20  <16 | 1,402,848,423  0  257,574,567  2,930,712 | 84.34  0  15.49  0.18 |
| Evapotranspiration  *January to August* | ≤ total precipitation  > total precipitation | 133,515,995  679,874,358 | 16.41  83.59 |
| Soil pH | 5-8  8-10  4-5  <4 | 304,832,964  8  358,057,749  74,052,041 | 41.36  0.00  48.59  10.05 |
| Soil Texture (% Clay) | 15-25  25-40  40-50  <15  >50 | 14,773,552  27,630,197  14,504,982  593,414  24,380,410 | 18.04  33.74  17.71  0.72  29.77 |
| # of Rainy Days (Disease Prone) | <30  30-60  60-75  75-90  90-105  105-120  120-135  135-150  >150 | 37,713,470  1,044,187,974  423,889,341  145,574,079  10,265,409  1,333,447  345,117  44,865  0 | 2.27  62.78  25.48  8.75  0.62  0.08  0.02  0.00  0 |
| Temperature (Disease Prone) | <15  15-20  20-25  25-35 | 966,291  259,538,987  712,819,098  690,029,325 | 0.06  15.60  42.85  41.48 |
| Land Cover | 11, 14, 18, 19, 21, 24  Other Categories | 182,019,050  646,127,839 | 21.98  78.02 |
